# Fountain of youth of pancreatic cancer cells: the extracellular matrix

**DOI:** 10.1038/s41420-017-0004-7

**Published:** 2018-01-10

**Authors:** Victoire Gouirand, Sophie Vasseur

**Affiliations:** 1 Centre de Recherche en Cancérologie de Marseille, U1068, Institut National de la santé et de la recherche médicale, Marseille, France; 2 Institut Paoli-Calmette, Marseille, France; 3 Unité Mixte de Recherche (UMR 7258), Centre National de la Recherche Scientifique (CNRS), Marseille, France; 4 Aix Marseille Université, Marseille, France

The microenvironment of tumors varies between cancer types, and its complex role in tumor propagation contributes to its importance in the field of tumor biology. Tumors with a dense microenvironment express aggressive characteristics that directly impact patient clinical outcomes. In pancreatic ductal adenocarcinoma (PDAC), tumor cells are surrounded by various cellular and acellular components including activated fibroblasts, immune cells, neurons, collagens, fibronectin, and hyaluronan, creating a fibrotic shield that impedes chemotherapeutics delivery to tumor cells^1–3^. Accordingly, Whatcott et al. have shown a negative correlation between PDAC-patient median survival and the collagen or hyaluronic acid enrichment of the primary tumor^4^. At the site of PDAC this fibrosis, or desmoplasia, often creates zones of low oxygen and nutrients resulting from poor vascularization^5^. Given these microenvironmental constraints and the resulting metabolic pressure, pancreatic tumor cells adapt their metabolism to benefit from available nutrient resources in order to survive and proliferate. This metabolic reprogramming leads to the emergence of aggressive tumor cells and invasive tumors, which are able to disseminate to distal organs including the liver and lung^6^.

**Fig. 1:**
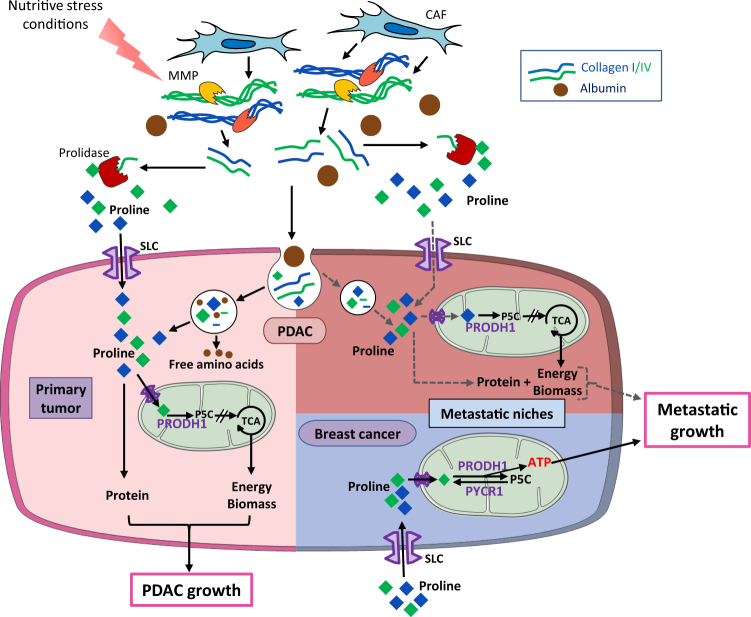
In PDAC tumor (light pink region), upon nutrient limitation, PDAC cells degrade collagen, which is produced by cancer-associated fibroblasts (CAFs) in the ECM, take up collagen fragments along with other proteins like albumin through macropinocytosis, and take up free proline either using the same process or through membrane transporters (SLC). Internalized vesicles fuse with lysosomes where collagen and albumin are degraded into free proline and free amino acids, all released in the cytosol. Proline is either incorporated into protein biomass or catabolized to fuel the tricarboxylic acid (TCA) cycle to promote PDAC cell survival and tumor growth. In liver metastasis (dark pink region) we can suspect the same metabolic process (shown by dark grey dotted arrows) as in primary PDAC for nutrient supply of growing metastatic niches. In breast cancer-derived lung metastases (blue region), proline catabolism supplies metastatic cells with adenosine triphosphate (ATP) to support their growth. *MMP* matrix metalloprotease; *SLC* solute carrier family

Recently published in *Nature Communications*^7^, our study shows an abundance of collagen types I and IV surrounding tumor cells among a variety of extracellular proteins and macromolecules contained in the PDAC microenvironment. Moreover, we observed an upregulation of matrix metalloproteases specific for collagen I and IV degradation as well as an increase of prolidase that hydrolyzes dipeptides with C-terminal proline or hydroxyproline residues, in the tumor compared to normal pancreas. Importantly, prolidase displayed an extracellular localization both in mouse and human PDAC sections. This suggests a potential breakdown of collagen to release proline that could be used as alternative nutrient source by PDAC tumor cells. Supporting this hypothesis, we have demonstrated an increase of proline dehydrogenase 1 (PRODH1), the key enzyme involved in proline catabolism, in mouse and human PDAC compared to normal pancreas. We then questioned whether PDAC cells could scavenge collagens for metabolic purposes and whether nutrient stress conditions promoted this scavenging. We found that PDAC cells increase their uptake of collagen I and IV proportionally to the intensity of the stress associated with glucose and glutamine deprivation. We discovered that upon glucose deprivation macropinocytosis is the main route PDAC cells use to engulf collagen and that blocking this process suppresses their survival. In vivo studies recently published in *Nature Medicine* by Davidson et al.^8^ showed that pancreatic tumors exhibit high levels of macropinocytosis and use this process to uptake albumin, the breakdown of which contributes to free amino-acid supply in the tumor. Hence, protein scavenging appears to be an important route for PDAC cells to satisfy their amino-acid demands and macropinocytosis is one of the main process K-ras mutant PDAC cells use to uptake surrounding macromolecules (Fig. 1)^9, 10^. Importantly, we have shown that, although PDAC cells take up collagen by macropinocytosis under glucose shortage, this process is not involved for internalization of collagens upon glutamine restriction or hypoxic stress, suggesting that PDAC cells are able to activate multiple mechanisms to internalize macromolecules depending the nature of the stress.

Our study is notable in its design and use of an experimental approach for in vitro collagen tracing; we cultured PDAC cells on labelled collagen matrix produced by fibroblasts previously supplied with labelled proline. We have shown that, when deprived of glucose or glutamine, tumor cells take up the labelled collagen matrix and extract proline from this collagen. To examine the role of proline in PDAC cells under glucose or glutamine-deprived cell culture contexts, we blocked proline degradation using the competitive inhibitor of PRODH1, deshydroproline. We observed a decrease of cell survival and proliferation, highlighting proline catabolism as a point of metabolic vulnerability that dictates PDAC cell growth. To better understand how proline helps tumor cell survive and proliferate, we carried out follow-up experiments to determine the metabolic contribution of proline in PDAC cells. Indeed, proline tracing in these cells revealed that proline carbons contribute to protein metabolic compartment. Also, we examined the fate of catabolized proline by metabolic tracing of U-^13^C-proline and found carbon labelling from proline degradation in all tricarboxylic acid (TCA) cycle intermediates in tumor cells under glucose and glutamine limitations at both normoxia and hypoxia. Inhibition of proline degradation not only reduced proline contribution to TCA cycle but also decreased the mitochondrial respiration of PDAC cells. Finally, to determine the contribution of proline to tumor growth, we chose to genetically invalidate PRODH1 in PDAC cells and challenge them to in vitro and in vivo tumorigenic assays. Indeed, PRODH1 inhibition impairs the clonogenic capacities of PDAC cells and drastically limits tumor size in vivo.

As a tumor embedded in a dense stromal reaction, we have shown that PDAC presents an important metabolic flexibility, which allows it to take advantage of the nutrient stores contained in the extracellular matrix (ECM). We propose that ECM-derived proline is one of the readily available substrates in tumor cores, which compensates for a lack in glutamine or glucose allowing tumor cell survival and proliferation under extreme metabolic challenge. Proline contribution to TCA metabolism, cellular respiration, or protein biomass identifies new metabolic pathways to be considered as pro-tumorigenic and which to date have been underestimated compared to glutamine or glucose metabolisms, especially in studies exploring PDAC metabolism. Another recent study published by Elia et al., in *Nature Communications*^11^, demonstrates that in the context of breast cancer, proline catabolism contributes to spheroidal growth through PRODH1 activity. Moreover, the pyrroline-5-carboxylate (P5C) reductase 1 responsible for the recycling of the P5C to proline appeared to contribute to the full activity of PRODH1 (Fig. 1). Elia et al. also showed that PRODH1 levels are higher in breast cancer metastases compared to primary breast tumors and that PRODH1 inhibition reduces the number of breast cancer-derived lung metastases without any impact on the primary tumors. In the context of breast cancer, the authors suggest that the inhibition of proline catabolism via PRODH1 targeting affects cancer cells' ability to establish metastasis most likely by impeding a phenotypic shift cancer cells need to operate at the first stage of micrometastasis formation once they have colonized the distant organ. In the context of metastatic PDAC, dynamic microenvironmental changes occur at the site of metastases; in liver metastases a size-dependent increase in myofibroblasts' recruitment and ECM components, both recapitulating the microenvironment of the primary tumor, has been reported^12^. These data are strengthened by others showing an identical repartition of acellular compounds between pancreatic primary tumor and metastases^4^. Then, one can hypothesize that PDAC cells could maintain the scavenging of collagen (among other nutrients) and its subsequent lysosomal breakdown in metastatic sites (Fig. 1). Targeting PDAC cells through the inhibition of their uptake of ECM could be a way to weaken aggressive PDAC cells with high metabolic demand both in the primary tumors as well as in metastatic niches. As long as the acquisition route relies on the macropinocytosis potential of cancer cells, pharmacological inhibition of this process in scavenging cells appears as a promising therapeutic window, as previously shown^8, 9^. However, our data show that a switch toward other endocytic processes occurs in PDAC cells, suggesting that a multiple targeting has to be considered to limit nutrient uptake in these cells.
